# Resistance Pattern of *Klebsiella pneumoniae* in Aseer Region, Saudi Arabia: A Ten-Year Hospital-Based Study

**DOI:** 10.3390/medicina60081344

**Published:** 2024-08-19

**Authors:** Saad Mohammed Alshehri, Naif Saud Abdullah, Abdullah Algarni, Ahmed Saad AlZomia, Mohammed Mushabub Assiry

**Affiliations:** 1 Preventive Medicine and Public Health Physician, Ministry of Health, Abha 62515, Saudi Arabia; 2 Community Medicine Consultant, Ministry of Health, Abha 62515, Saudi Arabia; 3 Family Medicine Consultant, Aseer Central Hospital, Abha 62515, Saudi Arabia; 4 Medical Intern, King Khalid University, Abha 62515, Saudi Arabia; 5 Senior Technician in the Department of Microbiology, Aseer Central Hospital, Abha 62515, Saudi Arabia

**Keywords:** antibiotic resistance, *Klebsiella pneumoniae*, trend shift, prevalence, factors, Saudi Arabia

## Abstract

*Background and Objectives:* The frequency of multidrug-resistant Klebsiella pneumoniae (MDRKP) has dramatically increased worldwide in recent decades, posing an urgent threat to public health. The aim of this study was to assess the extent of *K. pneumoniae* in the Aseer region and explore the corresponding antimicrobial resistance profile over the last ten years. *Materials and Methods:* A record-based retrospective study was conducted in a tertiary hospital during the period of 2013 to 2022. The study targeted laboratory samples taken from patients admitted to the hospital and sent for *K. pneumoniae* culturing. We included only samples taken from the patient and confirmed by the lab. Data were extracted using a pre-structured data extraction sheet to avoid data-collection bias and confirm the inter-rater precision. Statistical Package for Social Sciences (SPSS) version 26 was employed for statistical analysis. All relationships were tested using Pearson X^2^ test for categorical data or chi-square for linear trend for resistance rate over years. *Results*: We obtained 3921 samples of isolated *K. pneumoniae* out of 28,420 bacterial samples. The isolation rate began at 11.3% in 2013, decreased to 6.1% in 2016, and then increased to a peak of 16.3% in 2021, before slightly decreasing to 12.8% in 2022. In total, 23.7% of *K. pneumoniae* samples were identified in urine samples, 19% in sputum samples, 14% in wound samples, and 11.7% in blood samples. The overall antibiotic resistance rate of *K. pneumoniae* from 2013 to 2022 showed a significant increase, particularly during 2020 and 2021, before decreasing again in 2022. The resistance rate decreased from 22.2% in 2013 to 18.6% in 2016 and increased to 54.6% and 56.4% during 2020 and 2021, respectively (*p* = 0.039)*. Conclusions:* We observed a significant shift in *K. pneumoniae* resistance for some antibiotics during the study period, highlighting the urgent need for enhanced antimicrobial stewardship and infection-control measures.

## 1. Introduction

Klebsiella species are Gram-negative bacteria of the Enterobacteriaceae family, and include aerobic and facultative anaerobic species [1]. Klebsiella ozaenae, Klebsiella oxytoca, Klebsiella rhinoscleromatis, and *Klebsiella pneumoniae* (*K. pneumoniae*) are the most frequently isolated species [2,3]. These species produce plasmid-mediated extended-spectrum beta lactamases (ESBLs), which convert medications into inert forms [4]. *K. pneumoniae* is the most prevalent and pervasive species in nature, causing a variety of diseases in humans [4,5].

In the last decade, there have been increasing reports of antibiotic-resistant strains of this organism. Against this background, understanding the resistance patterns of *K. pneumoniae* worldwide would help in the prescription of appropriate antibiotics for several diseases [6,7]. This knowledge would also help hospital infection-control teams in decision-making processes for policies aimed at preventing the spread of multidrug-resistant strains [8,9].

Resistant bacterial infections have increased globally due to increases in morbidity and mortality rates caused by the largescale antimicrobial drug applications in clinical settings [10,11]. About one-third of all infectious diseases caused by Gram-negative bacilli are bloodstream infections, pneumonia, urinary-tract infections (UTIs), nosocomial infections, and community-acquired infections [12,13]. Resistant Klebsiella is an opportunistic pathogen extensively associated with antibiotic resistance [14].

Multidrug-resistant *K. pneumoniae* (MDRKP) has emerged as an important public health concern due to its dramatic increase in global incidence during the last few decades [15,16,17] and now represents one of the most well-known superbugs. Indeed, expression of the gene for colistin resistance, known as mcr-1; extended-spectrum beta-lactamases (ESBLs); and a number of carbapenemases is now a possibility for MDRKP. Additionally, MDRKP has become extensively drug-resistant (XDR) and multidrug-resistant (MDR) [18]. In the Aseer region, a previous study found that all *K. pneumoniae* isolates were resistant to commonly used antibiotics such as amoxicillin/clavulanate and ampicillin. Moreover, approximately 63% of the isolates were resistant to imipenem, and 57.4% were resistant to meropenem [19]. This result emphasizes the importance of conducting additional research and the urgent need for continuous monitoring.

The most appropriate method for determining population resistance patterns is to collect consecutive strains of an organism over a defined period and test them against all relevant antibiotics [20,21]. This epidemiological method of resistance typing involved testing 102 strains of *K. pneumoniae* against 13 antimicrobial agents and analyzing the data based on time and patient characteristics. This method provided more thorough data than the resistance patterns reported by many laboratories, which often provide resistance rates only for commonly used antibiotics [22,23]. The objective of the present study was to assess the extent of *K. pneumoniae* in the region of Aseer and explore the antimicrobial resistance profile of these isolates over the last ten years.

## 2. Methods

### 2.1. Study Design

A record-based retrospective study was conducted at a tertiary hospital in the Aseer region, the capital of the southern region of Saudi Arabia. This hospital serves as a reference and educational institution, receiving both direct and referred patients from across the Aseer region. All complete and eligible records from 2013 to 2022 were reviewed.

### 2.2. Sample Collection

This study focused on laboratory samples taken from patients admitted to the hospital and sent for *K. pneumoniae* culturing. Only the first samples from each patient, confirmed by the lab, were included. Various sample types were collected from multiple departments, including urine, sputum, wound, blood, abscess, swab, and bodily-fluid samples.

### 2.3. Bacterial Identification

Standard microbiological techniques were used for all samples, except for urine samples, which were cultured in cystine–lactose–electrolyte-deficient agar (CLED). All samples were incubated at 37 °C for 24 to 48 h on two media types: sheep blood agar and McConkey agar. A Vitek-2 automated system (bioMérieux, Marcy-l’Étoile, France) was employed for bacterial identification.

### 2.4. Antibiotic Susceptibility Testing

Antibiotic susceptibility was assessed using the N291, N292, and N204 cards on a Vitek-2 system. Additionally, a Microscan WalkAway automated system (Beckman Coulter, Brea, CA, USA) provided antibiotic susceptibility patterns using a Negative Breakpoint Combo 50 (NBC 50). Results were obtained within 24 to 48 h. The antibiotics tested included ciprofloxacin, Bactrim, cefepime, piperacillin/tazobactam, amoxicillin/clavulanate, levofloxacin, meropenem, amikacin, aztreonam, ertapenem, ampicillin/sulbactam, moxifloxacin, cefotaxime, imipenem, colistin, and tigecycline. Interpretation of the minimum inhibitory concentration (MIC) results followed the guidelines of the Clinical Laboratory Standards Institute (CLSI) [24].

### 2.5. Data Collection

Data were extracted using a pre-structured data extraction sheet to ensure inter-rater precision and prevent data collection bias. Extracted data included patient demographics, laboratory results, microbiology reports, and antimicrobial susceptibility testing for *K. pneumoniae*.

### 2.6. Ethical Considerations

Ethical approval was obtained from the institutional review board (IRB) with approval number REC-17-1-2024. Written consent was obtained from the administration of the hospital where the data were collected. Only the research team had access to the data, which were used solely for the present study. This study adhered to the principles of non-maleficence, ensuring no harm to the patients involved.

## 3. Data Analysis

Following data collection and evaluation, the Statistical Package for Social Sciences, version 26 (SPSS: An IBM Com SPSS Version 26, IBM Corp, Armonk, NY, USA) was used to process the data. Every statistical technique employed had a two-tailed alpha threshold of 0.05, and a *p*-value of less than or equal to 0.05 was deemed significant. In order to conduct descriptive analysis, the study variables were prescribed in terms of frequency distribution and percentage. All relationships were tested using the Pearson X^2^ test for categorical data or chi-square for linear trend for resistance rate over years. Line graphs were used to assess trend changes during the study years (2013 to 2022) in cases and resistance rates, while var charts were used to compare between ward and gender distributions. All extracted data were independently reviewed by two independent specialists to avoid any missing data or errors.

## 4. Results

**Figure 1:**
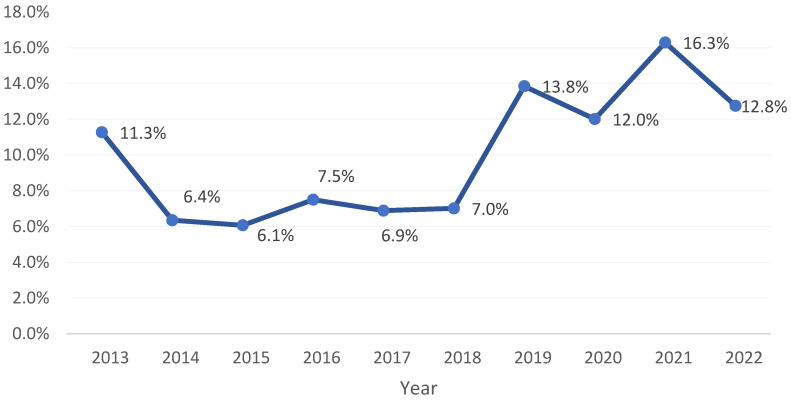
Isolation rate of *Klebsiella Pneumoniae* during the period 2013-2022, Aseer region, Saudi Arabia.

A total of 3921 samples of isolated *K. pneumoniae* out of 28,420 bacterial samples during the period 2013–2022 were collected in the Aseer region. The isolation rate was highest during the last 4 years (from 2019 to 2022) (Figure 1). In total, 2232 (56.9%) were from the general ward (GW) and 1689 (43.1%) were from the intensive care unit (ICU). Additionally, 2585 (65.9%) of the isolated samples were from males and 1336 (34.1%) were from females (Table 1).

**Figure 2:**
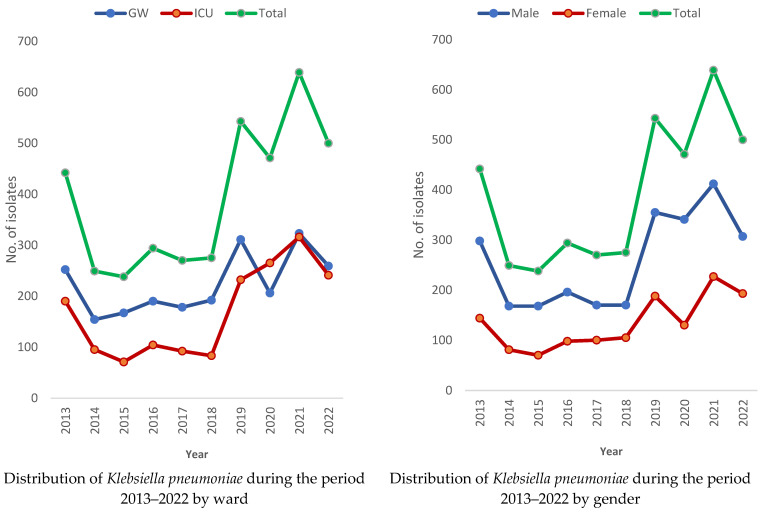
Distribution of *Klebsiella pneumoniae* cases during the period of 2013–2022 by ward in Aseer, Saudi Arabia.

**Figure 3:**
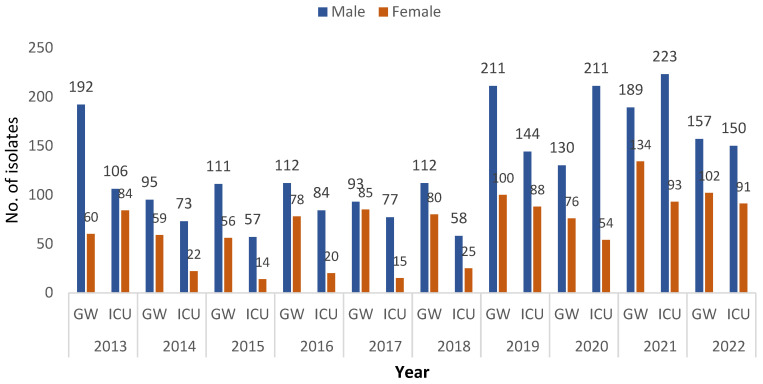
Number of *K. pneumoniae* isolates by case, gender, and ward during the period from 2013 to 2022.

The number of isolates of *K. pneumoniae* was higher among cases in the general ward, except during the last 4 years (2019–2022). However, the number of isolates remained higher among males than females (Figure 2 and Figure 3).

**Figure 4:**
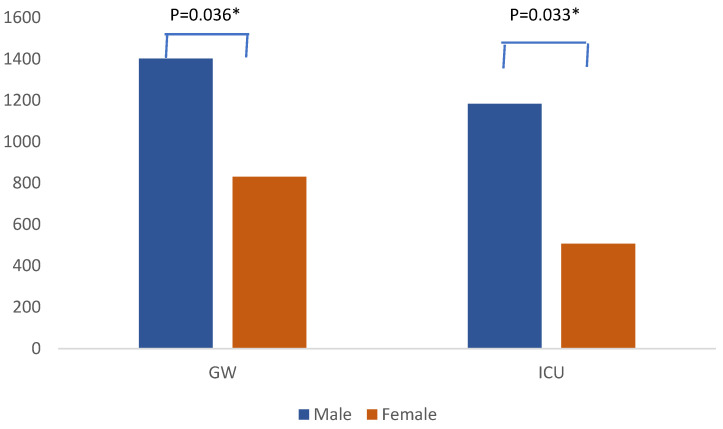
Distribution of *Klebsiella pneumoniae* isolates by case, gender, and ward. * *p* < 0.05 (significant).

*K. pneumoniae* isolates were most prevalent among males in both the general ward and the ICU during all years of the study. In total, 1402 isolates were reported among males in the general ward compared with 830 among females (*p* = 0.036), while 1183 isolates were reported among males in the ICU versus 506 isolates among females *(p* = 0.033) (Figure 4).

**Figure 5:**
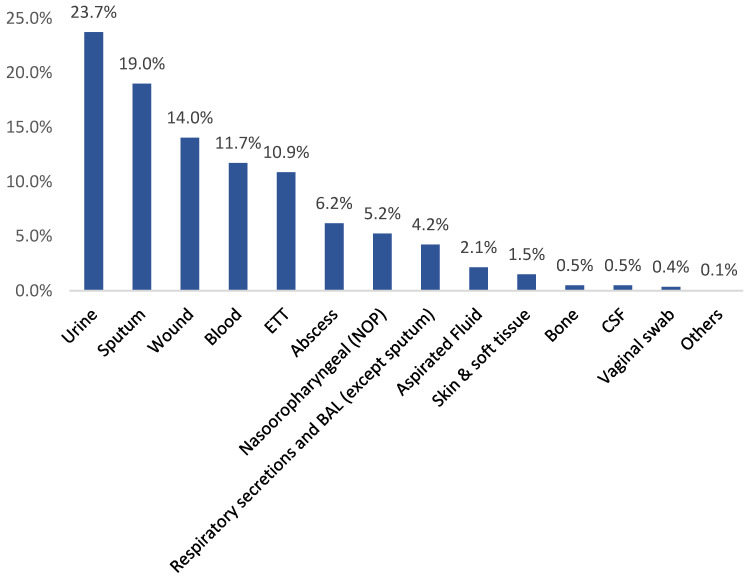
Distribution of *K. pneumoniae* isolates by source during the study period in the Aseer region.

Exactly 23.7% of the isolates were identified in urine samples, 19% in sputum samples, 14% in wound samples, 11.7% in blood samples, and 10.9% in endotracheal tube (ETT) samples, while 6.2% were isolated from abscesses (Figure 5).

Distribution of sample sources for *K. pneumoniae* isolates by ward: in the general ward, the most commonly reported sources of isolated *K. pneumoniae* were urine (30.3%), sputum (16.7%), and wounds (15.1%), compared with sputum (22%), blood (19.5%), ETT (16.1%), and urine (15%) in the ICU, with a recorded statistical significance *(p* < 0.05) (see Table 2).

The antibiogram pattern of *K. pneumoniae* among study cases from 2013 to 2022 in the Aseer region revealed a significant upward trend for *K. pneumoniae* resistance for many antibiotics, primarily in 2020 and 2021, e.g., ampicillin/sulbactam (from 0.2% to 34%), ertapenem (from 9.7% to 35.8%), meropenem (from 5.2% to 51.4%), moxifloxacin (from 13.3% to 20.6%), and levofloxacin (from 24.2% to 33.2%) (see Table 3).

**Figure 6:**
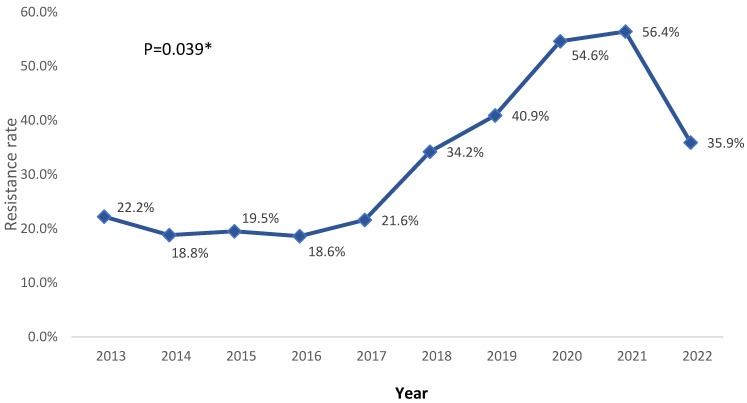
Overall antibiotic resistance rate trend of *K. pneumoniae* from 2013 to 2022. * *p* < 0.05 (significant).

The overall antibiotic resistance rate of *K. pneumoniae* from 2013 to 2022 showed a significant increase, particularly during 2020 and 2021, before decreasing again in 2022. The resistance rate decreased from 22.2% in 2013 to 18.6% in 2016 and increased to 54.6% and 56.4% during 2020 and 2021, respectively *(p* = 0.039) (Figure 6).

**Figure 7:**
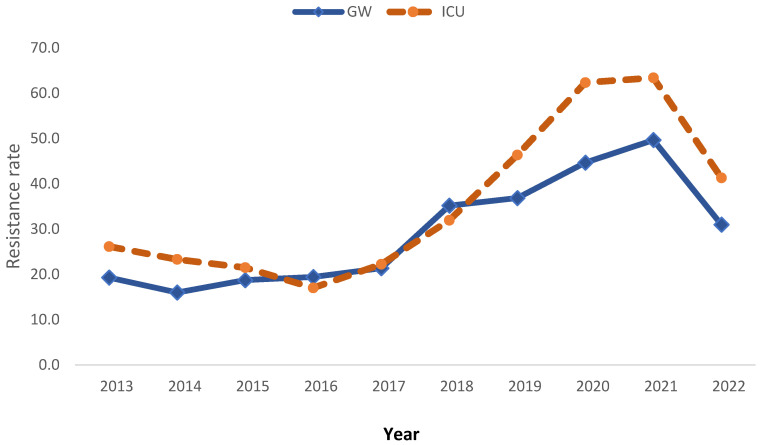
Overall antibiotic resistance rate trend of *K. pneumoniae* by ward from 2013 to 2022.

The data also show that the resistance rate was insignificantly higher among ICU cases than among general ward cases, mainly during last few years (2019 to 2022) (Figure 7).

**Figure 8:**
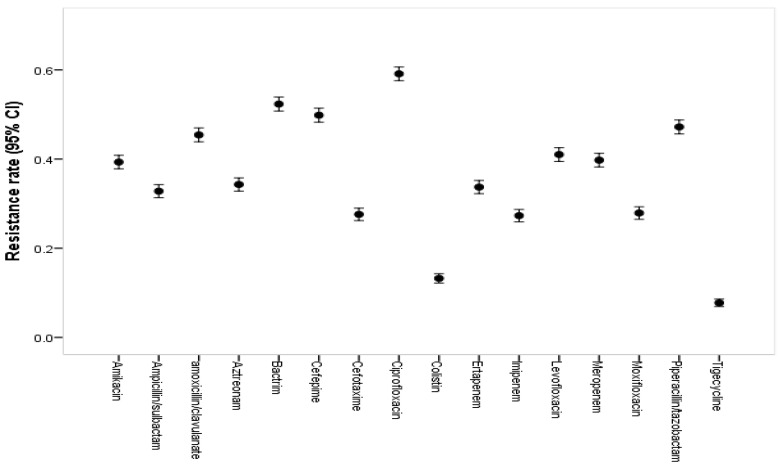
Resistance rates with 95% CIs for *K. pneumoniae* under different types of tested antibiotics.

The resistance rates and 95% CIs for *K. pneumoniae* under different types of tested antibiotics were determined. The highest overall resistance rates were identified for ciprofloxacin (59.1%; 95% CI, 57–62), Bactrim (52.3%; 95% CI 50.5–54), cefepime (49.8%; 95% CI 48–51.3), piperacillin/tazobactam (47.2%; 95% CI 45.2–49), amoxicillin/clavulanate (45.4%; 95% CI 43.9–47), levofloxacin (41%; 95% CI 39.4–42.9), and meropenem (39.8%; 95% CI 38.1–41.2). The lowest overall resistance rates were identified for cefotaxime (27.6%; 95% CI 26–29), imipenem (27.3%; 95% CI 26–29), colistin (13.2%; 95% CI 12–15), and tigecycline (7.8%; 95% CI 7.1–9) (see Figure 8).

## 5. Discussion

Overall, the emergence and dissemination of multidrug-resistant *K. pneumoniae* strains represent significant public health risks at the global level [25,26]. The findings from this study can help workers understand the resistance patterns of *K. pneumoniae*, facilitating empirical, evidence-based clinical choices for treatment and infection-control measures. Finally, this research will help workers devise strategies to overcome the challenge of antimicrobial resistance, which will eventually improve patient outcomes and support the rational management of *K. pneumoniae* infections in clinical settings.

According to the present study, the number of *K. pneumoniae* infections has gradually risen over the past several years. The percentage of isolated *K. pneumoniae* samples increased from 11.3% in 2013 to 16.3% in 2021. This result is comparable to the findings of Jalal N.A. et al. [27], who discovered that the prevalence of *K. pneumoniae* grew rapidly from 7.7% in 2011 to a peak of 25.9% in 2020. The increased percentages of isolated *K. pneumoniae* samples during 2020 and 2021 in our study were similar to the results of other research, which found a significant increase in CRKP incidence during the COVID-19 pandemic [28]. Among the study samples, the average infection rate with *K. pneumoniae* was 13.7%, which is lower than the rates previously estimated in several publications. Infections with *K. pneumoniae* were found to be prevalent in Asser and Bisha, Saudi Arabia, at rates of 39% and 18.6%, respectively [29,30]. Saudi Arabia’s *K. pneumoniae* infection prevalence, however, agrees with global data (18.8 to 87.7% in Asia and 5 to 35% in Western countries) [31]. On average, 65.9% of *K. pneumoniae* isolates from male patients were found to represent the majority of isolates in the present study. This finding is consistent with the outcomes of other studies, which found most *K. pneumoniae* isolates to come from male patients [27,30,32]. The present study also revealed that 56.9% of the isolates were from cases in the general ward, rather than the ICU.

Additionally, the present study showed that urine, sputum, wound, blood, and ETT samples provide the majority of *K. pneumoniae* isolates. This result underscores the dominance of UTIs and respiratory-tract infections among the study cases. This result does not differ significantly from previous studies, which reported blood samples to have the highest rates, followed by sputum [19,27]. The current findings are based on the observation that *K. pneumoniae* is a primary cause of bloodstream and respiratory-tract infections. This factor should be considered when diagnosing respiratory infections [33,34].

The present study also showed a significant upward shift in antibiotic resistance from 22.2% in 2013 to 56.4% in 2021 and decreasing to 35.9% in 2022, with an average overall resistance rate of 32.2%. Ciprofloxacin, Bactrim, cefepime, piperacillin/tazobactam, and amoxicillin/clavulanate offered the highest resistance (nearly half of the sample or more). Cefotaxime, imipenem, colistin, and tigecycline exhibited the lowest overall resistance rates. Among the studied antibiotics, ampicillin offered the highest resistance rate, with an average of 97.6%, according to Jalal NA et al. [27]. Other research conducted in Aseer and Medina observed a resistance rate of 100% and 99.9%, respectively, for ampicillin, which is much higher than the estimated rate in the present study [19,29]. Our research also revealed that *K. pneumoniae*’s resistance to third- and fourth-generation cephalosporins increased dramatically, with rates for cefotaxime and cefepime rising to 46% and 68.1% in 2021, respectively. This result is consistent with earlier Saudi Arabian reports showing an increase in *K. pneumoniae* antibiotic resistance to cephalosporins [19,29]. Ethiopia, China, and other countries worldwide have also reported similar findings, which suggests a global increase in *K. pneumoniae*’s resistance to third- and fourth-generation cephalosporins [26,35,36]. Much lower resistance was reported in India (2018) [37], where 12.5% were classified as susceptible, 7.5% were resistant, 7.5% were MDR, and 62.5% were XDR. In the 2018 group, the antibiotic combinations of amoxicillin/clavulanic acid (90%), ciprofloxacin (100%), piperacillin/tazobactam (92.5%), and cefoperazone/sulbactam (95%) were shown to provide the highest resistance percentages. No strains were susceptible in 2022. However, 21.4% of the strains were classed as resistant, 7% as MDR, and 93% as XDR, reflecting the same upward change in the *K. pneumoniae* resistance rate. Gram-negative bacteria produce infections that can be treated with carbapenem, which is a powerful antibiotic with a long history of use as a primary treatment option for infections induced by bacteria that produce ESBLs. However, resistance to carbapenems is showing serious signs of increasing, which could lead to worldwide problems in the future [38]. We also evaluated the resistance profile for meropenem in this study. The results indicated a notable increase in the resistance rate from 5.2% in 2013 to 51.4% in 2022. For ertapenem, we observed an increase from 9.7% in 2013 to 35.8% in 2022. There have also been reports of a rapid increase in carbapenem-resistant Enterobacteriaceae (CRE) both locally and globally [19,39].

Notably, the present study’s focus on a single tertiary hospital in the Aseer region may limit the generalizability of our findings to other regions or healthcare settings. Moreover, the specific impact of the COVID-19 pandemic on the increases in *K. pneumoniae* infections was not investigated. This could have provided further insights into the observed trends. Additionally, the study did not include detailed clinical data on patient outcomes or characteristics such as age and comorbidities, which would have strengthened our analysis of antibiotic resistance impacts on clinical outcomes.

Future research addressing these limitations will help achieve a more comprehensive understanding of resistance patterns and their impacts on patient care. Despite these limitations, the present study provides valuable insights into the prevalence and resistance patterns of K. *pneumoniae* in the Aseer region, contributing to the global understanding of this significant public health threat.

## 6. Conclusions and Recommendations

In summary, we observed a discernible increase in *K. pneumoniae* cases over the course of the investigation. The rates of resistance against nearly all of the drugs under investigation also increased notably. The reported *K. pneumoniae* antibiotic resistance is a serious concern that should be closely observed and managed. The toxicity of last-resort drugs and the absence of viable treatment choices create difficulties in handling “superbugs” such as XDR Klebsiella. Thus, to stop and control the development of antimicrobial resistance in *K. pneumoniae* and other bacteria resistant to multiple drugs, it is imperative to implement active antimicrobial stewardship programs, as well as intensive teaching programs for clinical pharmacists and physicians.

## Figures and Tables

**Table 1:** Demographic distribution of *Klebsiella pneumoniae* during the period 2013–2022, Aseer region, Saudi Arabia.

Year	Total		Ward				Gender			
			GW		ICU		Male		Female	
	No	%	No	%	No	%	No	%	No	%
2013	442	11.3%	252	57.0%	190	43.0%	298	67.4%	144	32.6%
2014	249	6.4%	154	61.8%	95	38.2%	168	67.5%	81	32.5%
2015	238	6.1%	167	70.2%	71	29.8%	168	70.6%	70	29.4%
2016	294	7.5%	190	64.6%	104	35.4%	196	66.7%	98	33.3%
2017	270	6.9%	178	65.9%	92	34.1%	170	63.0%	100	37.0%
2018	275	7.0%	192	69.8%	83	30.2%	170	61.8%	105	38.2%
2019	543	13.8%	311	57.3%	232	42.7%	355	65.4%	188	34.6%
2020	471	12.0%	206	43.7%	265	56.3%	341	72.4%	130	27.6%
2021	639	16.3%	323	50.5%	316	49.5%	412	64.5%	227	35.5%
2022	500	12.8%	259	51.8%	241	48.2%	307	61.4%	193	38.6%
Total	3921	100.0%	2232	56.9%	1689	43.1%	2585	65.9%	1336	34.1%

**Table 2:** Distribution of sample source for *Klebsiella pneumoniae* isolates by ward.

Source	Ward				*p*-Value
	GW		ICU		
	No	%	No	%	
Urine	677	30.3%	253	15.0%	0.004 *
Sputum	373	16.7%	372	22.0%	0.036 *
Wound	337	15.1%	213	12.6%	0.048 *
Blood	129	5.8%	330	19.5%	0.001 *
ETT	154	6.9%	272	16.1%	0.039 *
Abscess	214	9.6%	28	1.7%	0.069
Aspirated fluid	72	3.2%	12	0.7%	0.534
Nasooropharyngeal (NOP)	145	6.5%	60	3.6%	0.339
Respiratory secretions and BAL (except sputum)	49	2.2%	117	6.9%	0.417
Skin and soft tissue	47	2.1%	12	0.7%	0.661
Vaginal swab	11	0.5%	3	0.2%	0.886
Bone	18	0.8%	1	0.1%	0.869
CSF	5	0.2%	14	0.8%	0.874
Others	1	0.0%	2	0.1%	0.996

*p*: Exact probability test, * *p* < 0.05 (significant).

**Table 3:** Antibiogram pattern of *K. pneumoniae* among study cases from 2013 to 2022 in the Aseer region.

Antibiotics	Year-Wise Prevalence (%) of Resistant *K. pneumoniae*																				Overall		*p*-Value for Trend
	2013		2014		2015		2016		2017		2018		2019		2020		2021		2022				
	No	%	No	%	No	%	No	%	No	%	No	%	No	%	No	%	No	%	No	%	No	%	
Ciprofloxacin	261	59.0%	115	46.2%	109	45.8%	140	47.6%	132	48.9%	140	50.9%	301	55.4%	349	74.1%	482	75.4%	289	57.8%	2318	59.1%	0.058
Bactrim	174	39.4%	68	27.3%	96	40.3%	108	36.7%	131	48.5%	169	61.5%	317	58.4%	325	69.0%	434	67.9%	230	46.0%	2052	52.3%	0.049 *
Cefepime	136	30.8%	107	43.0%	101	42.4%	107	36.4%	74	27.4%	107	38.9%	329	60.6%	316	67.1%	435	68.1%	242	48.4%	1954	49.8%	0.046 *
Piperacillin/tazobactam	80	18.1%	55	22.1%	88	37.0%	105	35.7%	79	29.3%	113	41.1%	290	53.4%	319	67.7%	453	70.9%	269	53.8%	1851	47.2%	0.043 *
Amoxicillin/clavulanate	219	49.5%	81	32.5%	78	32.8%	61	20.7%	79	29.3%	59	21.5%	265	48.8%	306	65.0%	415	64.9%	218	43.6%	1781	45.4%	0.058
Levofloxacin	107	24.2%	60	24.1%	68	28.6%	84	28.6%	67	24.8%	125	45.5%	231	42.5%	305	64.8%	395	61.8%	166	33.2%	1608	41.0%	0.047 *
Meropenem	23	5.2%	13	5.2%	32	13.4%	61	20.7%	58	21.5%	84	30.5%	268	49.4%	318	67.5%	445	69.6%	257	51.4%	1559	39.8%	0.002 *
Amikacin	179	40.5%	70	28.1%	68	28.6%	76	25.9%	30	11.1%	92	33.5%	201	37.0%	252	53.5%	395	61.8%	179	35.8%	1542	39.3%	0.158
Aztreonam	187	42.3%	24	9.6%	0	0.0%	2	0.7%	10	3.7%	100	36.4%	241	44.4%	276	58.6%	356	55.7%	149	29.8%	1345	34.3%	0.096
Ertapenem	43	9.7%	14	5.6%	0	0.0%	3	1.0%	47	17.4%	108	39.3%	231	42.5%	291	61.8%	406	63.5%	179	35.8%	1322	33.7%	0.006 *
Ampicillin/sulbactam	1	0.2%	7	2.8%	9	3.8%	28	9.5%	17	6.3%	156	56.7%	255	47.0%	274	58.2%	369	57.7%	170	34.0%	1286	32.8%	0.001 *
Moxifloxacin	59	13.3%	23	9.2%	0	0.0%	0	0.0%	71	26.3%	101	36.7%	181	33.3%	244	51.8%	312	48.8%	103	20.6%	1094	27.9%	0.039 *
Cefotaxime	42	9.5%	76	30.5%	55	23.1%	10	3.4%	37	13.7%	47	17.1%	148	27.3%	236	50.1%	294	46.0%	137	27.4%	1082	27.6%	0.087
Imipenem	34	7.7%	20	8.0%	40	16.8%	59	20.1%	54	20.0%	52	18.9%	147	27.1%	154	32.7%	334	52.3%	177	35.4%	1071	27.3%	0.053
Colistin	6	1.4%	7	2.8%	0	0.0%	16	5.4%	6	2.2%	23	8.4%	85	15.7%	119	25.3%	199	31.1%	58	11.6%	519	13.2%	0.117
Tigecycline	20	4.5%	8	3.2%	0	0.0%	13	4.4%	43	15.9%	28	10.2%	63	11.6%	31	6.6%	46	7.2%	52	10.4%	304	7.8%	0.328

The *p*-values for trends were calculated using a chi-squared test. A *p*-value of less than 0.05 indicates an increase in the resistance trend. * *p* < 0.05 (significant).

## Data Availability

The original contributions presented in the study are included in the article, further inquiries can be directed to the corresponding author.
